# Recovery of ζ-chain expression and changes in spontaneous IL-10 production after PSA-based vaccines in patients with prostate cancer

**DOI:** 10.1038/sj.bjc.6600039

**Published:** 2002-01-21

**Authors:** N Meidenbauer, W Gooding, L Spitler, D Harris, T L Whiteside

**Affiliations:** University of Pittsburgh Cancer Institute, W1041 Biomedical Science Tower, 211 Lothrop Street, Pittsburgh, Pennsylvania, PA 15213-2582, USA; Department of Pathology, University of Pittsburgh School of Medicine, W1041 Biomedical Science Tower, 211 Lothrop Street, Pittsburgh, Pennsylvania, PA 15213, USA; Department of Otolaryngology, University of Pittsburgh School of Medicine, W1041 Biomedical Science Tower, 211 Lothrop Street, Pittsburgh, Pennsylvania, PA 15213, USA; Jenner Biotherapies, Tiberon, California, CA 94920, USA; Department of Medicine, Thomas Jefferson University, Philadelphia, Pennsylvania, PA 19107, USA

**Keywords:** PSA, vaccination, prostate cancer, T-cell functions, immune monitoring

## Abstract

Circulating T lymphocytes of patients with prostate cancer have been reported to have functional deficits, including low or absent ζ-chain expression. To determine whether these functional impairments could be reversed by prostate specific antigen-based vaccination therapy, 10 patients treated with recombinant human prostate specific antigen plus GM-CSF and eight others receiving prostate specific antigen plus oil emulsion in two pilot clinical trials were evaluated prior to and after vaccination for several immunologic end points, including ζ-chain expression and cytokine production by circulating T cells as well as the frequency of T cells able to respond to prostate specific antigen in ELISPOT assays. The flow cytometry assay for ζ-chain expression was standardized to allow for a reliable comparison of pre- *vs* post-vaccination samples. Prior to therapy, the patients were found to have significantly lower ζ-chain expression in circulating CD3^+^ cells and a higher percentage of ζ-chain negative CD3^+^ and CD4^+^ cells than normal donors. The patients' peripheral blood mononuclear cells spontaneously produced more IL-10 *ex vivo* than those of normal controls. After vaccination, recovery of ζ-chain expression was observed in 50% of patients in both clinical trials. Also, spontaneous IL-10 secretion by peripheral blood mononuclear cells decreased following immunotherapy in patients treated with prostate specific antigen and GM-CSF. The frequency of prostate specific antigen-reactive T cells was detectable in 7 out of 18 patients vs 4 out of 18 patients prior to vaccination. Only one of 18 patients was a clinical responder. The vaccine had stimulatory effects on the patients' immune system, but post-vaccine immune recovery could not be correlated to progression-free survival in this small cohort of patients with prostate cancer.

*British Journal of Cancer* (2002) **86**, 168–178. DOI: 10.1038/sj/bjc/6600039 www.bjcancer.com

© 2002 The Cancer Research Campaign

## 

In patients with advanced cancer, functional impairments of circulating and tumour-infiltrating lymphocytes have been frequently reported. For example, lymphocytes of cancer patients often show reduced proliferative and/or cytotoxic responses, have an altered cytokine profile and typically exhibit reduced/absent expression of TcR-associated signal-transduction molecules (reviewed Alving *et al*, 1993). Expression of one of these molecules, the ζ-chain, was shown to be decreased in T cells of patients with a variety of solid tumours, including renal, colorectal, gastric, pancreatic, cervical and prostate cancer (Whiteside, 1999). Decreased ζ-chain expression in circulating T cells before therapy appeared to correlate with the presence of advanced disease in patients with cancer (Matsuda *et al*, 1995; Zea *et al*, 1995). Furthermore, low or absent ζ-chain expression in tumour-infiltrating T-cells was identified as an independent and significant predictor of shorter survival in patients with advanced oral carcinoma (Reichert *et al*, 1998). Decreased ζ-chain expression reflects the diminished functional competency of T cells. Both tumour-infiltrating and circulating T cells in patients with cancer have been found to express less or no ζ and to have functional defects (Rabinowich *et al*, 1998; Reichert *et al*, 2000).

It has been reported that non-specific immunotherapy with cytokines or adoptively-transferred autologous lymphocytes plus IL-2 results in a recovery of ζ-chain expression in peripheral T cells of some of the treated patients (Bukowski *et al*, 1998; Gratama *et al*, 1999; Kono *et al*, 1998; Tartour *et al*, 1995). To investigate the possibility that active specific immunotherapy can normalize ζ-chain expression in T cells of patients with prostate cancer and lead to improved T-cell functions, we studied a cohort of patients receiving PSA-based vaccines. We have previously shown that the administration of a PSA-based vaccine resulted in the generation of PSA-reactive T cells in this cohort of patients with surgically incurable prostate cancer (Meidenbauer *et al*, 2000).

In the present study, we evaluated lymphocytes, which were obtained from the same patients prior to and after vaccination, for changes in ζ-chain expression in parallel with monitoring the frequency of PSA-reactive T cells in the peripheral circulation and cytokine profiles of the T cells. Our results indicate that in some of these patients, PSA-based vaccination resulted in the restoration of impaired ζ-chain expression and generation of PSA-reactive circulating T cells.

## MATERIALS AND METHODS

### Prostate specific antigen (PSA)

Recombinant human PSA was produced in insect cells infected with baculovirus containing the open reading frame for PSA. Sequence analysis of the PCR amplification product revealed a single nucleotide difference of C to A at the position 643 compared with the published sequence (Lundwall and Lilja, 1987), corresponding to an amino acid change from Pro to Glu. Due to the baculovirus cleavage site, the amino terminus contained two extra amino acids, Asp and Leu. Culture supernatants containing recombinant (r) PSA were purified by affinity chromatography, using a murine monoclonal anti-PSA antibody (clone HB 8527, purchased from ATCC, Rockville, MD, USA) in a good-manufacturing practice (GMP) facility at the TSI Washington Laboratories (Rockville, MD, USA). This recombinant PSA was used to prepare the vaccine.

### Liposomal PSA vaccine (JBT 1001)

Recombinant PSA at the concentration of about 100 μg ml^−1^ was added to the lyophilized lipids and incubated at 4°C. The liposomes were diluted in 20 mM Tris-glycine-buffer-150 mM sodium chloride, pH 7.4 and centrifuged at 30 000 **g** for 30 min to remove unencapsulated PSA. Liposomes were manufactured under GMP conditions, as previously described (Alving *et al*, 1993; Wassef *et al*, 1994). PSA-containing liposomes (1.2 ml) were placed in 2 ml vaccine vials (Wheaton, Millville, NJ, USA) and designated as JBT 1001 vaccine. Prior to administration to patients, JBT 1001 was tested for pyrogenicity, safety, residual solvents immunogenicity as well as sterility. Toxicology studies in rabbits were also performed.

### Patients

Eighteen patients with prostate carcinoma, who participated in two pilot vaccination trials, are evaluated in this immunological study. The first pilot trial was performed in 11 patients treated by subcutaneous (s.c.) administration of JBT 1001 with locally-injected GM-CSF (125 μg daily for 5 days). In the second trial, 11 patients received intramuscular (i.m.) injections of JBT 1001 emulsified in light mineral oil as adjuvant. To enter these pilot trials, patients had to meet the following eligibility criteria: histologically documented prostate cancer; surgically incurable disease, an ECOG performance status of 0 and 1 and an estimated life expectancy of more than 6 months. Furthermore, they had to have completed surgery or chemotherapy/ radiation at least 4 or 6 weeks, respectively, before starting the vaccine regimen. Hormonal treatment was permitted. All patients had a hematocrit of at least 32%, adequate hepatic function (bilirubin and SGOT <3×upper limit of normal) and renal function (BUN and creatinine ⩽1.5×upper limit of normal). The blood PSA-values were required to be <50 ng ml^−1^, and all the patients had the ability to be sensitized against dinitrofluorobenzene (DNFB). Steroid therapy, active second malignant disease (except basal squamous cell carcinoma of the skin) or cerebral metastases were exclusion criteria. All patients gave informed consent, and the study was conducted at Lankenau Hospital (Wynnewood, PA, USA) following approval by the Institutional Review Board. An investigational new drug (IND) application was on file with FDA. Prior to vaccination, the patients had a complete physical examination, chest X-ray, bone scan and CT-scan of the abdomen and pelvis. Peripheral blood was obtained for CBC, chemistry panel, PSA and immunological studies on days 0, 30, 60 and 90. Additional blood samples were drawn 60 days after the delivery of vaccination boosts. Patients were sensitized to DNFB prior to entry on the protocol. The delayed-type hypersensitivity (DTH) response to human PSA was tested on day 90. Table 1

**Table 1:**
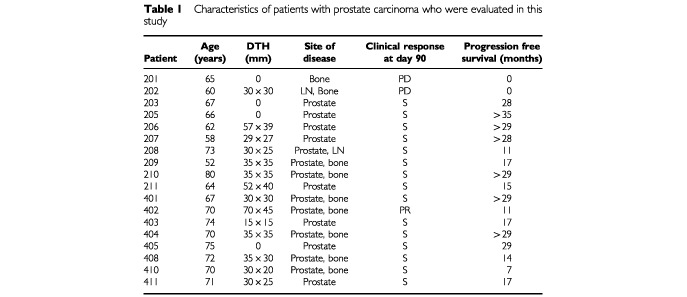
Characteristics of patients with prostate carcinoma who were evaluated in this study

provides an overview of the patient characteristics.

The 18 of 22 vaccinated patients, who were included in this immunologic evaluation, remained on study at least until day 90. Four of the 22 patients did not remain in the study until day 90 and, therefore, were not included in the immunologic evaluation (two with disease progression, two withdrew because of personal reasons). Seven normal donors age-matched with the patients (median age 73 years; range from 61 to 80 years) as well as 12 normal donors recruited from among the laboratory personnel (median age 33 years; range from 30 to 49 years) were included in the study.

### Vaccination schema

In the trial with GM-CSF as adjuvant (the 200 series), patients received 1 ml JBT 1001 containing PSA (100 μg) and lipid A (200 μg) on days 0, 30 and 60 subcutaneously (s.c.). One hundred and twenty-five μg of recombinant GM-CSF (Immunex Corp., Seattle, WA, USA) was injected s.c. into the vaccination site on days 0–4, 30–34, and 60–64.

In the vaccination trial using oil emulsion (the 400 series), JBT 1001 was emulsified with light mineral oil to form oil-in-water emulsion and delivered intramuscularly (i.m.). Each ml of the vaccine contained 90 μg PSA, 180 μg lipid A and 0.1 ml of mineral oil emulsified in a syringe fitted with a 3-way stopcock. The immunization schedule was as described above.

In both trials, patients with stable disease at day 90 after vaccination received booster vaccinations every 60 days until disease progression.

### Collection and storage of blood specimens

Prior to vaccine administration, patients underwent leukapheresis, and peripheral blood mononuclear cells (PBMC) were harvested on Ficoll-Hypaque, washed, counted, resuspended in the freezing medium and cryopreserved in liquid N_2_ in cryovials, each containing 20–30×10^6^ cells. Following the initial vaccination sequence (days 0, 30, 60) peripheral blood specimens were obtained on days 90 (Post), 150 (Boost no. 1), 210 (Boost no. 2), 270 (Boost no. 3) and 330 (Boost no. 4). The cryopreserved PBMC were thawed just prior to the initiation of the assays, diluted into warm RPMI-medium (Gibco Life-Technologies, Grand Island, NY, USA) supplemented with 20% (v/v) foetal calf serum (FCS; Gibco Life-Technologies), and washed twice with RPMI medium. Viability was checked using a Trypan blue dye and was found to be >90% in all instances.

### Determination of ζ-chain expression by two-colour flow cytometry

All assays were performed on cryopreserved and thawed PBMC. Aliquots of cells (2.5×10^6^ ml^−1^ in 200 μl of PBS) were dispensed into tubes and incubated for 30 min with 5 μl aliquots of one of the following antibodies: CD3-FITC, CD4-FITC, CD8-FITC or isotype IgG as control (all purchased from Becton Dickinson, San Jose, CA, USA). After the incubation period, the cells were washed twice with PBS and then fixed with 0.25% (w/v) paraformaldehyde for 10 min at room temperature. The cells were again washed twice with PBS and then once with cold saponin (0.1% w/v in PBS+0.1% w/v BSA) solution. The cells were then permeabilized for 30 min on ice in 100 μl of the saponin solution. Aliquots (10 μl) of TcRζ–PE or isotype-IgG (Immunotech, Marseilles, France) were added at the same time. Following the incubation period, the cells were washed twice with saponin and then once with PBS. Flow cytometry analysis was performed immediately on FACScan (Becton Dickinson). At least 10 000 events were acquired for data analyses.

The analysis was performed using the Lysis II program. The lymphocyte gate was established based on FCS/SCC. Lymphocytes were then backgated to CD3^+^, CD4^+^ or CD8^+^ populations and their mean fluorescence intensity (MFI) determined. In addition to MFI, the percentage of ζ-negative cells was determined. Based on the cutoff level established by using the MFI values obtained with the isotype control, it was possible to classify the T cell subsets as ζ-positive and ζ-negative. Pre- and post-therapy samples were always tested in the same assay.

Aliquots of PBMC obtained from one normal donor were cryopreserved and tested each time the assay was run to control for inter-assay variability. To further standardize the assay and to determine the intra-assay and the inter-individual variability of ζ-chain expression in T cells, PBMC were obtained from six normal male donors and were tested in triplicate in assays performed on different days. To estimate intra-individual (biologic) variability, PBMC samples were obtained from six male donors at three different times separated by at least 1 month, and the cryopreserved serial samples were tested together in one assay.

### Separation of PBMC into CD4*^+^* and CD8*^+^* T cells

PBMC were separated into CD4^+^ and CD8^+^ T cells by positive immunoselection, using magnetic beads (MiniMacs, Miltenyi Biotec, Auburn, CA, USA) according to the manufacturer's recommendations. The purity of the selected CD4^+^ or CD8^+^ T-cell fractions was checked by flow cytometry.

### Generation of dendritic cells (DC)

Leukapheresis products obtained from each patient prior to entry on the protocol were processed as previously described (Meidenbauer *et al*, 2000). Dendritic cells were generated from cryopreserved PBMC in 7 day cultures, using IL-4 and GM-CSF as described by us before (Meidenbauer *et al*, 2000). On day 6, 10 μg ml^−1^ of purified human PSA (Cortex Biochem, San Leandro, CA, USA) or 10 μg ml^−1^ of ovalbumin (Sigma) were added to the DC culture for a period of 12–16 h. For some patients, non-pulsed DC were used as control DC. The cells were then harvested by incubating them with cold Hanks' Balanced Salt Solution (Gibco Life Technologies) for 15–30 min. After two washing steps, the DC were adjusted to the required concentrations, based on the content of large cells and used as APC.

### ELISPOT assay for IFN-γ production

The ELISPOT assay was performed exactly as previously described by us (Meidenbauer *et al*, 2000). Briefly, T lymphocytes (CD4^+^ or CD8^+^) separated by positive selection on immunobeads were placed in wells of 96-well ELISPOT plates at 10^5^ cells per well. Then, autologous DC pulsed with PSA or ovalbumin (as a control antigen) were added. Control wells containing PBMC and DC not pulsed with any antigen were also set up. The assay was usually run in triplicate or quadruplicate. After 24 h incubation of the plates in a humidified atmosphere of CO_2_ in air at 37°C, the plates were developed to visualize the spots as previously described (Meidenbauer *et al*, 2000). The spots were counted by computer-assisted image analysis (ELISPOT 4.143, Zeiss, Jena, Germany). The number of specific spots was calculated by subtracting control values (spots counted for DC not pulsed with PSA and co- incubated with PBMC) from experimental values. The number of spots in control wells was either 0 or 1 for all specimens in this series. The assay was standardized as described earlier (Asai *et al*, 2000; Meidenbauer *et al*, 2000), and its CV was 15% with *n*=19. The sensitivity of the assay was estimated to be 1/10^5^ cells (Asai *et al*, 2000).

### Cytokine production assays

After thawing, 100 μl aliquots of PBMC were plated at the concentration of 1×10^6^ cells ml^−1^ into wells of 96-well U-bottom plates (Linbro, ICN Biomedicals, Auroram CA, USA). One hundred μl aliquots of RPMI^+^ 10% FCS (Gibco Life Technologies) with or without PHA (20 μg ml^−1^) (Sigma) were added to each well. After 48 h of incubation in a humidified atmosphere of 5% CO_2_ in air at 37°C, the supernatants were removed, centrifuged once to remove cell debris and stored at −80°.

### ELISA for IL-2 and IL-10

IL-2 and IL-10 levels in the supernatants were determined by ELISA (R&D systems) according to the manufacturer's recommendations. Each sample was tested in duplicate, and the standard dose-responsive curves were established using the WHO cytokine standards.

### Statistical analysis

ζ-chain expression for patients was compared to that of normal healthy controls by the exact Wilcoxon test. If multiple assay results were available for normal controls, the median was used. Fisher's exact test was used to compare proportions of response between the different treatment groups. The overall change with treatment was tested with a signed rank test applied to the differences between pre-and-post treatment ζ-chain expression. Individual patients were classified as either increasing, not changing or decreasing the ζ-chain expression in T cells with treatment. An individual patient increase or decrease in ζ-chain expression was defined as exceeding a 95% confidence bound calculated from repeated measurements of normal controls who were assumed to represent normal biologic variability between measurements. Correlations between immunologic parameters and clinical parameters were calculated using Spearman's rank test.

## RESULTS

### Standardization of the assay for ζ-chain expression

ζ-chain expression in T lymphocytes was evaluated by flow cytometry, measuring both mean fluorescence intensity (MFI) and the percentage of ζ-chain-negative cells in the population (Kuss *et al*, 1999). Cryopreserved cells were used based on earlier comparisons made between fresh and cryopreserved PBMC (i.e., each sample was split and tested fresh as well as after cryopreservation). These comparisons indicated no significant differences in ζ-chain expression between the samples (data not shown). Inter-assay variability was determined for both MFI and the percentage of ζ-chain-negative cells, using cells of one normal donor which were cryopreserved, thawed and tested for ζ-chain expression every time the assay was performed on 10 different days. The MFI values for ζ-chain were: 332±32 (s.e.m.) in CD3^+^ T cells, 423±35 in CD4^+^ T cells and 298±20 in CD8^+^ T lymphocytes. Because of this substantial inter-assay variability, we routinely test all pre- and post-vaccine samples of one patient in the same assay. The mean percentage of ζ-chain-negative T cells was less than 1% in this donor's cells, too low to be reliably used for determination of the assay variability.

To establish intra-assay variability for ζ-chain expression, six PBMC samples, each obtained from a different donor, were tested in triplicate in flow cytometry assays performed on different days. The results of triplicate determinations for each donor were very tight (i.e., the intraclass correlations were >0.8 for MFI), indicating that intra-assay variability was excellent for both MFI and the per cent of ζ-negative cells.

**Figure 1:**
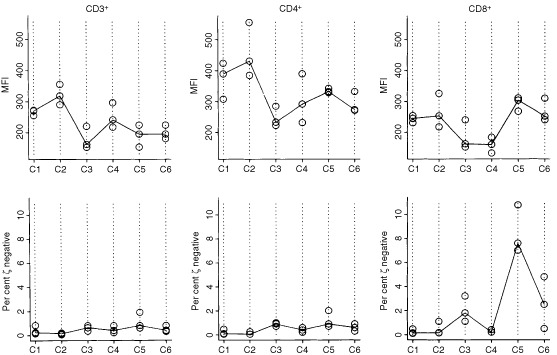
Intra-individual variability of MFI for ζ-chain expression and the per cent of ζ-chain-negative cells in CD3^+^, CD4^+^ and CD8^+^ T cell subsets obtained from six normal donors (C1–6). The samples were drawn at three different time points 1 month apart. PBMC were cryopreserved and tested in the same assay to establish the biologic variation within each individual evaluated over a 3 month period. The solid line connects the median values. Of note, the percentages of ζ-chain-negative T cells are very low in normal controls, except for CD8^+^ T cells in C5.

In addition, cells of all six donors were repeatedly obtained at three time points separated by 1 month, and at each time point were tested together in one assay in order to determine biologic (intra-individual) variability in ζ-chain expression (Figure 1

). This was important, because the patients' cells were sampled at different time points, even when the pre- and post-vaccine samples were tested in the same assay. The intra-individual (biologic) variations for three different samples obtained from the same normal donor over a period of 3 months and tested in the same assay were considerable, as shown in Figure 1. We assumed that they would be equally substantial for the patients' cells. Therefore, to define a significant change in ζ expression (i.e., a change that was larger than expected and, therefore, not due to assay variability) for an individual, we set a cutoff value for MFI of the ζ-chain as well as for the percentage of ζ-chain-negative cells. This cutoff value was defined to be greater than half the width of the 95% confidence interval determined for intra-individual variability as described above. Thus, a change of 62 MFI units for ζ in CD3^+^ cells, 113 units for ζ in CD4^+^ cells and 72 units for ζ in CD8^+^ cells detected in an individual sample was considered to be significantly greater than normal biologic variability for this individual. For the percentage of ζ-chain-negative T cells in CD3^+^, CD4^+^ and CD8^+^ populations, these values were 0.7, 0.7 and 2.6%, respectively. Patients were scored as responders in terms of ζ-chain expression when a significant change, as described by the above criteria, in the MFI or the percentage of ζ-negative cells occurred following vaccination.

### Differences in ζ expression between patients and controls

**Figure 2:**
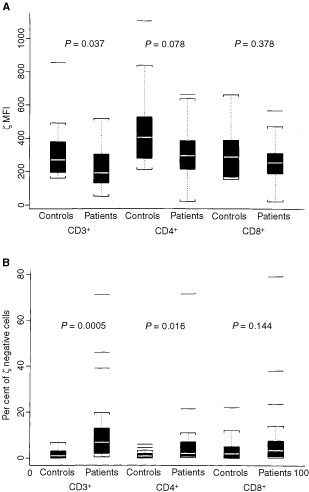
Comparisons of the ζ-chain expression in T cells and T-cell subsets between patients and healthy controls are presented as box- whisker plots. The white bar represents the median; the box represents the interquartile range; the whiskers represent 1½ times the interquartile range; and the single lines show the outliers. Nineteen healthy controls were compared with 18 patients with prostate cancer. (**A**), MFI for ζ-chain expression and (**B**), percentages of ζ-chain-negative cells in patients and healthy controls are shown. The *P* values were calculated by the exact Wilcoxon test.

The analyses for ζ-chain expression were performed in CD3^+^, CD4^+^ and CD8^+^ lymphocytes obtained from patients and controls (Figure 2

). Although the control group included seven age-matched and 12 younger individuals, no significant difference in ζ expression was observed between the older and younger donors in this and other studies performed in our laboratory (data not shown). The two subgroups were, therefore, combined into one control group, and ζ-chain expression was compared in T cells of patients and normal donors. Both the MFI for ζ and per cent ζ-negative cells were significantly lower for CD3^+^ T cells in patients with prostate cancer than in controls (*P*=0.0374). As a group, these patients with prostate cancer had a significantly decreased percentage of ζ-positive CD3^+^ and ζ-positive CD4^+^ T cells, but not of the CD8^+^ T cells, when compared to normal donors (Figure 2). It should be noted that in controls, only a very small proportion of ζ-negative T cells was detected (usually <1%), while in the patients, the percentage of ζ-negative T cells varied broadly from none to more than 70% (Figure 2b).

**Figure 3:**
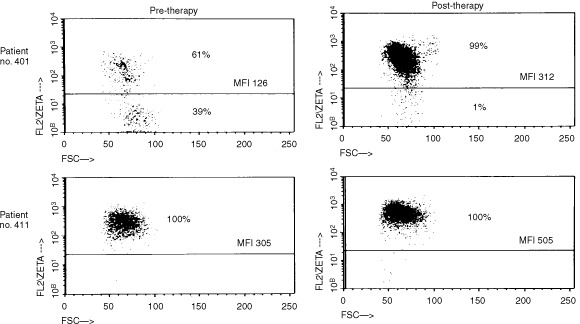
Flow cytometry dot plots for ζ-chain expression in two representative patients with prostate carcinoma. The gate was set on CD3^+^ T cells. In patient no. 401, ζ-chain-positive and ζ-chain-negative populations of T cells are present. In patient no. 411 practically all T cells express ζ-chain. After vaccination, nearly all T cells of patient no. 401 express ζ-chain, and MFI for ζ is increased in T cells of both patients.

Also, as illustrated in Figure 3

, two distinct populations of T cells were identifiable in the circulation of some of the patients: ζ-positive and ζ-negative (e.g., patient no. 401). In other patients, nearly all T cells were ζ-positive (e.g., patient no. 411), a situation comparable to what is typically observed in T cells of normal donors. The percentage of ζ-negative cells allowed us a better discrimination than MFI between these two groups of patients. For this reason, we selected to present the flow cytometry data both as MFI for ζ expression and as percentages of ζ-negative cells (Figure 2a and b).

### Changes in expression of ζ-chain after vaccination

**Figure 4:**
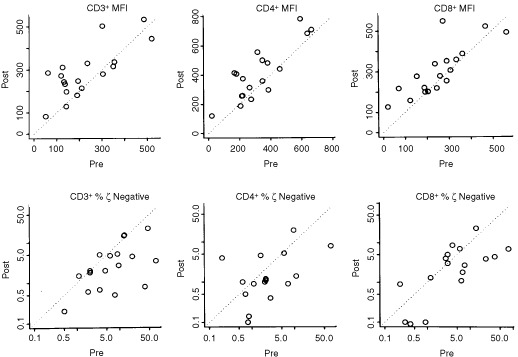
Treatment-related changes in ζ-chain expression in T cells of all the patients. The upper charts summarize changes in MFI and the lower charts, changes in the percentage of ζ-negative T cells. The diagonal dotted lines indicate no change in zeta expression from pre- to post-therapy.

The comparison of pre- and post-therapy samples, using both parameters (MFI and the percentage of ζ-chain-negative T cells) and applying the criteria established to rule out biologic variability, indicated that up-regulation of ζ expression occurred in T cells of a proportion of patients (Figure 4

). In six patients (no's. 205, 206, 210, 401, 402 and 403) a significant decrease in the percentage ζ-chain-negative CD3^+^ cells was accompanied by a significantly increased MFI for the ζ-chain after vaccination (Table 2

**Table 2:**
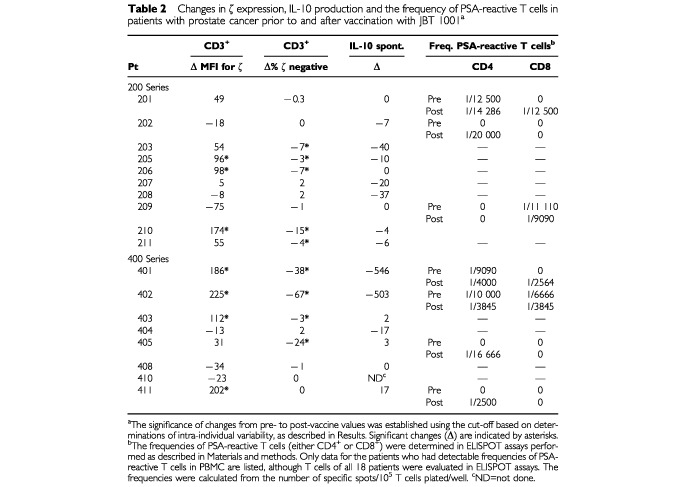
Changes in ζ expression, IL-10 production and the frequency of PSA-reactive T cells in patients with prostate cancer prior to and after vaccination with JBT 1001^a^

). These changes were observed in both CD4^+^ and CD8^+^ T cell sub-populations (Figure 4).

**Figure 5:**
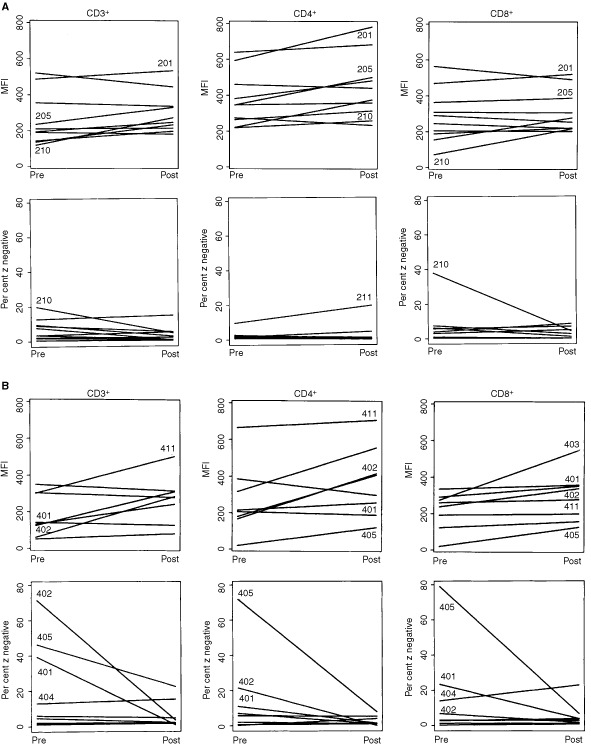
ζ-chain expression prior to and after therapy with PSA-based vaccines. (**A**) The data for the patients in the 200 series (PSA+GM-CSF). (**B**) The data for the patients in the 400 series (PSA emulsified in mineral oil). The numbers identify patients with most pronounced pre- to post-therapy changes in ζ-expression. Note that the changes in the 400 series are generally more pronounced. Comparison between pre and post treatment by 2-tailed signed rank test showed significant changes (*P*<0.05) only for the patients in the 400 series. MFI of ζ-chain expression increased for CD3^+^, CD4^+^ and CD8^+^ cells. The comparison of percentage ζ-chain-negative cells showed a significant decrease (*P*<0.05) only in the CD3^+^ cells.

When the patients included in the 200 and 400 series were separately examined for ζ expression in T cells prior to and after vaccination therapy, it appeared that the more extensive changes were observed in the 400 series (Figure 5b

). However, when pre- and post-vaccine changes in the percentage of ζ-negative T cells were compared, 50% of patients in both series showed significantly increased ζ-chain expression (Table 2). Also, the patients with the highest per cent of ζ-negative T cells prior to therapy showed the largest per cent decrease after treatment.

### Frequency of PSA-reactive T cells

To determine the frequency of PSA-reactive T cells in the peripheral circulation, PBMC were enriched in CD4^+^ and CD8^+^ cells and then tested in 24 h ELISPOT assays. In order to correlate ELISPOT results with ζ-chain expression, the frequency of PSA-responsive T cells was measured directly in PBMC without *in vitro* stimulation (IVS), since *in vitro* culture could restore the impaired ζ-chain expression (Rabinowich *et al*, 1996). As reported previously by us, the assay is very sensitive, capable of detecting 1 out of 100 000 positive cells and has a negligible non-specific background, when PBMC are used as responders (Asai *et al*, 2000).

Although the observed frequencies of PSA-reactive T cells in the circulation of patients were generally low, as previously reported by us (Meidenbauer *et al*, 2000), in 7 out of 18 patients a detectable number of precursor T cells responsive to PSA was present (Table 2). In three of these seven patients, the frequency of PSA-reactive T cells increased after vaccination. In 3 out of 18 patients (nos. 201, 401 and 402), we found a detectable number of PSA-reactive T cells before treatment (see Table 2). In age- and sex-matched normal donors, only one out of seven tested samples had a detectable frequency of PSA-reactive T cells (1 out of 10 000 in CD4^+^ T cells).

It is also interesting to note that 3 out of 4 of the patients who had negative DTH responses to PSA on day 90 (nos. 201, 202 and 405), had detectable but very low frequencies of PSA-reactive T cells in the circulation after the vaccine administration.

### Cytokine production assays

**Figure 6:**
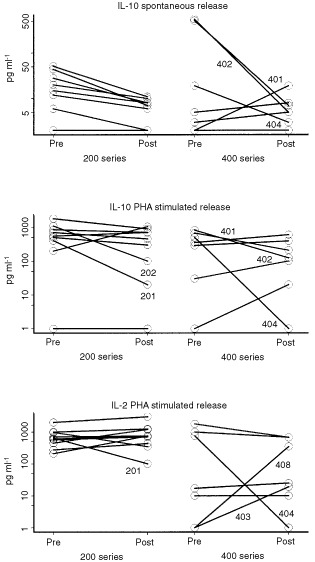
Cytokine secretion by the patients' PBMC. Either spontaneous or PHA-stimulated secretion of IL-10 or IL-2 by PBMC, were determined by ELISA in the supernatants after 48 h culture. Note that patients nos. 401 and 402 have high pre-vaccination levels of IL-10, which decreased after treatment. Only changes in IL-10 spontaneous release for patients in the 200 series are statistically significant at *P*<0.01.

To get additional information about the immune status of the patients, levels of IL-2 and IL-10 secreted by PBMC spontaneously or after stimulation with the mitogen, PHA, were measured in 43 patient samples and six control PBMC specimens obtained from sex- and age-matched normal donors. Spontaneous IL-2 production was detected in 5 out of 18 pre-therapy samples by ELISA (data not shown). In contrast, spontaneous IL-10 (>5 pg ml^−1^) production by PBMC was observed in 11 out of 18 pre therapy samples (Figure 6

). Only 1 out of 6 normal PBMC spontaneously produced IL-10, while the cytokine profile of the patients was skewed toward IL-10 production prior to vaccination.

PHA-stimulated production by PBMC of IL-10 and IL-2 was evaluated as a measure of general immunocompetence. No significant vaccine-related changes were observed in stimulated IL-10 or IL-2 release (Figure 6), although in the 200 series, a trend toward lower IL-10 production and higher IL-2 release was observed after vaccination.

Spontaneous production of IL-10 by the patients' PBMC was found to be significantly decreased after vaccination only in the 200 vaccine series (Figure 6; *P*<0.01). In the two patients (nos. 401 and 402) whose PBMC spontaneously secreted high levels of IL-10 (>500 pg ml^−1^) prior to treatment, the level of IL-10 secreted by PBMC dropped to 10 pg ml^−1^ after vaccination (Figure 6). Interestingly, in these two patients, low ζ-chain expression observed prior to therapy increased significantly after treatment, and an increase in the number of PSA-reactive T cells was observed as well.

### Correlations between the immunological parameters and clinical outcome

Attempts were made to correlate immunological parameters with the clinical outcome. Progression free survival (PFS) was selected as a clinical end point, because 16 out of 18 patients were still alive (March, 2000), and among them only one partial response was observed.

In this cohort of patients, no correlation between DTH in response to PSA after therapy and PFS was evident. Also, no correlations could be established between PFS and ζ-chain expression before therapy, changes in ζ-chain expression after therapy or an increased frequency of PSA-reactive T cells. However, the three patients (nos. 401, 402, 411), who mounted a significant T-cell responses after treatment with the vaccine, as determined by ELISPOT and a DTH response to PSA, and the only patient (no. 402) with a documented PR (improvement in bone scintigraphy) also showed significant increases in ζ-chain expression after therapy.

## DISCUSSION

Two small pilot vaccination trials with PSA performed in patients with advanced prostate cancer allowed us an opportunity to examine changes in several immunologic variables during therapy. The study focused on T-cell responses in order to assess the impact of therapy on both PSA-specific and non-specific immunity. The rationale for the study was based on the hypothesis that successful vaccination is associated with upregulation of functions in immune cells, particularly their PSA-related activity. Thus, the presence of functional, activated T cells, which are thought to be largely responsible for anti-tumour effects, is a prerequisite for the efficacy of the vaccine.

One goal of this study was to determine whether ζ-chain expression in circulating T cells could be altered by the vaccine. We expected that the PSA vaccine plus oil emulsion or GM-CSF as adjuvants may have general immunostimulatory effects, leading to increased ζ-expression in T cells. Immunotherapy with cytokines or autologous lymphocytes was previously reported to increase ζ-chain expression (Bukowski *et al*, 1998; Gratama *et al*, 1999; Kono *et al*, 1998; Tartour *et al*, 1995). After the PSA vaccine, recovery of ζ-(i.e., decreased per cent of ζ-negative cells) was observed in 50% of our patients regardless of the adjuvant used. These data suggest that the vaccine containing GM-CSF was not more immunostimulatory than that containing PSA + oil emulsion.

Recent studies have considered the possibility that cancer-related signalling defects in lymphocytes, e.g., low or absent ζ-chain expression, encountered in patients with advanced disease might contribute to limited responses or the lack of responses frequently seen with immunologically-based therapies (Bukowski *et al*, 1998; Gratama *et al*, 1999; Kuss *et al*, 1999; Rabinowich *et al*, 1996). Our patients with prostate cancer evaluated prior to vaccination, showed significantly decreased expression of the ζ-chain in circulating T lymphocytes compared to normal controls, a finding similar to that previously reported by Healy *et al* (1998). However, not all T cells had low ζ and not all patients with prostate cancer had T cells with decreased ζ expression (Figure 3). In a subset of these patients, the percentage of ζ-chain-negative T cells was significantly increased in the peripheral circulation compared to healthy volunteers, who had very few ζ-chain-negative T cells, except in one person's CD8^+^ cells (Figure 1). It appears that a gradient of ζ-chain loss exists in the patient population, ranging from normal ζ expression to its absence in only some or most of circulating T cells. The per cent of ζ-negative cells in patients was significantly decreased among CD3^+^ and CD4^+^, but not CD8^+^ cells, although some individual patients had very high proportions of ζ-negative CD8^+^ cells never seen in controls. Because ζ expression is lower in CD8^+^ than CD4^+^ T cells (Kuss *et al*, 1999) and is also more variable in CD8^+^ T cells of normal donors and patients alike, the observed lack of significance for CD8^+^ cells is attributed to a small sample size.

Several distinct mechanisms seem to contribute to a rapid turnover of ζ (reviewed by Whiteside, 1999) and may be responsible for differences in ζ expression in T cells of patients with cancer reported in the literature (Bukowski *et al*, 1998; Cardi *et al*, 1997; Deakin *et al*, 1999; Healy *et al*, 1998; Kuss *et al*, 1999). Although the mechanism(s) responsible for ζ-down-regulation in cancer remains unclear, it appears to be tumour-related (Gastman *et al*, 1999; Reichert *et al*, 1998; Whiteside, 1999). The degree of ζ impairment is greater in TIL than in PBL in individual patients (Matsuda *et al*, 1995; Rabinowich *et al*, 1996). Normal T cells co-incubated with tumour cells or their supernatants down-regulate ζ-chain expression (Dworacki *et al*, 2001; Maccalli *et al*, 1999). Recent data indicate that tumour-derived circulating factors, such as 14 kD protein described by Taylor *et al*. (2001) or tumour associated HLA-class I molecules (Maccalli *et al*, 1999) could mediate this effect. The tumour microenvironment is rich in H_2_O_2_ or NO, which are secreted by activated macrophages *in situ* (Kono *et al*, 1996) or activated granulocytes in the circulation of patients with cancer (Schmielau and Finn, 2001). Oxygen metabolites have been shown to interfere with T cell functions and to down-regulate ζ (Kono *et al*, 1996). Also, the presence of chronic antigenic stimulation in cancer patients could lead to activation-induced cell death (AICD) and depletion of tumour-specific T cells by apoptosis (Dworacki *et al*, 2001; Horiguchi *et al*, 1999). We previously reported that the ζ-chain is a target for caspases in apoptosing lymphocytes, an observation which links the loss of ζ with apoptosis (Gastman *et al*, 1999). The low frequency of PSA-specific T cells in the circulation of our patients seems to be consistent with this last hypothesis.

The biologic and clinical significance of the ζ-chain recovery in cancer patients treated with immunotherapy is also not clear. While previous studies demonstrated a significant relationship between ζ expression in T cells and patient survival (Reichert *el al*, 1998; Zea *et al*, 1995), the relevance of ζ expression in T cells of patients with prostate cancer or its clinical and prognostic importance is unknown. In this study, no significant correlations could be established between ζ-chain expression before or after therapy and DTH responses to PSA or an increase in the frequency of PSA-reactive T cells after therapy and progression-free survival. Nevertheless, some interesting insights were obtained. Three patients (all in the 400 series) showed significant up-regulation of ζ expression as well as a substantial increase in PSA-reactive T cells after therapy (nos. 401, 402 and 411). All three had positive DTH to PSA. The only patient who had a documented PR to the vaccine (no. 402) was in this subgroup. The two patients with PD had relatively normal ζ expression before and after therapy. Among 15 patients with s.d., there were eight with PFS >20 months and seven with PFS <20 months. In the former group, 6 out of 8 (75%) showed significant ζ-up-regulation *vs* 3 out of 7 (42%) in the latter. While this difference is not statistically significant, it suggests that ζ expression may have some relationship to clinical responses, although larger studies will be necessary to determine its clinical significance.

The frequency of PSA-reactive precursor cells in the patients' circulation after therapy ranged from 1 out of 2500 to 1 out of 20 000, as assessed by ELISPOT and was comparable to the frequencies reported by Lewis *et al*. (2000) for cancer patients treated with peptide-based vaccines. But in 11 out of 18 patients no detectable PSA-reactive T cells were present either before or after vaccination, although all but four of the patients were able to respond to PSA by DTH after vaccination. This implies that functions of T cells, as measured in *in vitro* assays, were suppressed in patients with prostate cancer. One clue may be the relatively high levels of IL-10, considered to be an immunosuppressive cytokine (Moore *et al*, 1993) and an independent prognostic factor in solid tumours (De Vita *et al*, 2000), which was spontaneously produced by patients' PBMC. After therapy, spontaneous production of IL-10 decreased in general, but most dramatic decreases were seen in the two patients (nos. 401 and 402) who had higher frequencies of PSA-specific T cells after vaccination therapy.

In summary, our results indicate that a PSA vaccine was able to restore impaired ζ-chain expression and to generate specific T cells in some patients with advanced prostate cancer. Although a correlation between clinical outcome and immunological endpoints could not be established in this small cohort of patients, monitoring of ζ-chain expression, the frequency of PSA specific T cells and the cytokine profile may be able to define patients who are capable of mounting a cellular response against the tumour. Other studies seeking a correlation between immunologic and clinical responses to vaccines in patients with prostate cancer report conflicting results (Lodge *et al*, 2000; Murphy *et al*, 2000). In one of these studies the percentage of ζ-positive cells within the CD3^+^ population was identified as one of 10 variables predictive of clinical responses in patients with metastatic disease (Murphy *et al*, 2000). The findings we report provide an encouragement that by using immune markers such as ζ expression, it might be possible in the future to single out patients who are most likely to benefit from vaccine-based therapies.
